# Brush Use in Lot-Fed Cattle Shows Continued Use and Positive Behaviour

**DOI:** 10.3390/ani15010044

**Published:** 2024-12-27

**Authors:** Emma J. Dunston-Clarke, Catherine Stockman, Josie Sinclair, Teresa Collins

**Affiliations:** 1 Food Futures Institute, School of Agricultural Sciences, College of Environmental and Life Sciences, Murdoch University, Perth 6150, Australia; emma.dunston@murdoch.edu.au; 2 Food Futures Institute, School of Veterinary Medicine, College of Environmental and Life Sciences, Murdoch University, Perth 6150, Australia; catherine.stockman@murdoch.edu.au (C.S.);

**Keywords:** environmental enrichment, cattle brush, feedlot, ethogram, affective state, temperament

## Abstract

Feedlot cattle were assessed for the consistency of enrichment brush use, higher incidence of natural behaviour, and less agonistic or abnormal behaviour compared to cattle without the brush. Cattle were assigned to one of two treatment pens, (1) access to a vertical grooming brush (EB; *n* = 89) or (2) no access control (CON; *n* = 80), for a period of 107 days. A Principal Component (PC) analysis was used on pen-side demeanour scores. Statistical analysis on pen-side behaviour and demeanour PCs tested for impact of treatment, day, and time. Frequency of brush use and the duration of each cow–brush interaction did not decrease over the assessment period. Self-grooming and allogrooming did not differ between treatments and were similar in frequency to brush grooming, resulting in total grooming being higher in the EB treatment group (*p* < 0.05). Cattle in the EB pen were scored as more content (PC 2; *p* < 0.05) and sociable (PC 3; *p* < 0.01), while CON cattle were scored as more anxious (PC 3; *p* < 0.05). Overall, this study suggests that the provision of a brush for enrichment enhanced lot-fed cattle wellbeing and permitted prolonged engagement, making it an effective enrichment device.

## Simple Summary

This study assessed the behavioural and production responses of lot-fed cattle when provided with enrichment in the form of a vertical grooming brush (EB) compared to cattle with no enrichment brush (CON) for a period of 107 days. Frequency of brush use and the duration of each cow–brush interaction did not decrease over the assessment period. Self-grooming and allogrooming did not differ between treatments and was similar in frequency to brush grooming, resulting in the total grooming being higher in the EB treatment group. Cattle housed in the EB pen were observed to engage in play behaviours more frequently than CON cattle. Aggression and abnormal behaviours were minimal and not different between treatments. Cattle in the EB pen were scored as more *content* and *sociable*, while CON cattle were scored as more *anxious*. Weight gain and temperament were not different between treatments. Overall, the study suggests that the provision of a brush enrichment for lot-fed cattle enhances their wellbeing and permits prolonged engagement, making it an effective enrichment device.

## 1. Introduction

The impact of intensive management systems on cattle welfare has had increasing focus both in society and by producers [1,2]. Emphasis is not only placed on health and access to suitable resources, but also the freedom to display natural behaviour [3]. Cattle welfare can be targeted through many different aspects of a feedlot management system. One of these is through the provision of enrichment, aiming to provide animals with autonomy and the ability to satisfy natural, species-specific behaviour [4]. This will likely reduce the negative psychological and physiological impacts typical of intensive management systems [5]. Context-specific research benefits the study of enrichment as it accounts for the influence of the management system on any potential livestock welfare parameters.

The efficacy of different forms of enrichment varies significantly depending on the type and context of the enrichment. In dairy and beef systems, enrichment research has focused on visual enrichments, such as mirrors [6] and images of familiar conspecifics [7], ropes for oral interaction [8,9], auditory enrichment through music [10], nutritional enrichment through pasture access [5,11], scent enrichment [12], and grooming brushes [12,13,14,15,16,17]. The economic viability and welfare benefits of such enrichment depend on an animal’s habituation to it, as there can be a progressive decrease in interaction with the enrichment over time [18]. Cattle habituate rapidly to many forms of enrichment [8,12,19,20]; however, studies have shown that calves, lactating dairy cows, and beef cattle consistently use enrichment brushes [12,13,15,21,22], leading to brushes becoming a popular choice of enrichment in cattle production systems [23].

Brush enrichment for cattle can provide monetary benefits by reducing their destructive behaviours toward infrastructure [9] and decreasing agonistic encounters that may lead to carcass bruising [14]. Temperament can influence productivity [24,25]; however, the response of cattle temperament to brush enrichment has not been examined in the context of a feedlot management system. Conflicting findings on live weight gain in response to brush provision also need further investigation [9,14]. Although enrichment may not improve productivity, it is noteworthy that it does not diminish it, as maintaining productivity ensures the sustainability and economic viability of the production system. However, enrichment is important to enhance natural behaviour indicative of good welfare, which can indirectly support long term productivity and farm sustainability and mitigate societal concerns for cattle in feedlot production systems [26,27].

Quantitative behaviour, such as increased grooming frequency, longer grooming duration, increased bouts of play, and reduced physiological stress responses have been demonstrated to occur in cattle in response to brush enrichment [13,26,27,28,29]. Emotional responses to brush enrichment have been limited to assessment of ear position and body posture, with a study finding that positive emotional responses increased with brush enrichment [30]. Demeanour scoring is a novel assessment of behavioural expression that is derived from Qualitative Behavioural Assessment [31]. It involves real-time pen-side assessment of behavioural expression by one or two observers using a specific list of terms. It has been successfully used to assess behavioural expression within live export and feedlot contexts [32,33]. Demeanour, combined with quantitative behaviour scoring, permits a more holistic evaluation of behaviour and welfare states and therefore would be a useful tool in understanding the benefits of enrichment [34,35].

The current study extends the scope of previous work by determining if brush use is sustained for the duration of an industry-standard feeding regime of approximately 100 days. In addition, it will assess both live weight and temperament change at the beginning and end of the feeding period. This study will examine not only quantitative behavioural changes to brush enrichment but also the more novel assessment of demeanour. The culmination of the behavioural and production assessments made will provide further insight into the value of brush provision to both the producer and the cattle within an Australian feedlot production system.

## 2. Materials and Methods

All procedures were approved by the Murdoch University Animal Ethics Committee (Permit No. R3106/19). The study took place from February to May 2021 during the first 107 days that the cattle were in a commercial feedlot located in Lake Preston, Western Australia.

### 2.1. Animals and Feedlot Design

**Figure 1:**
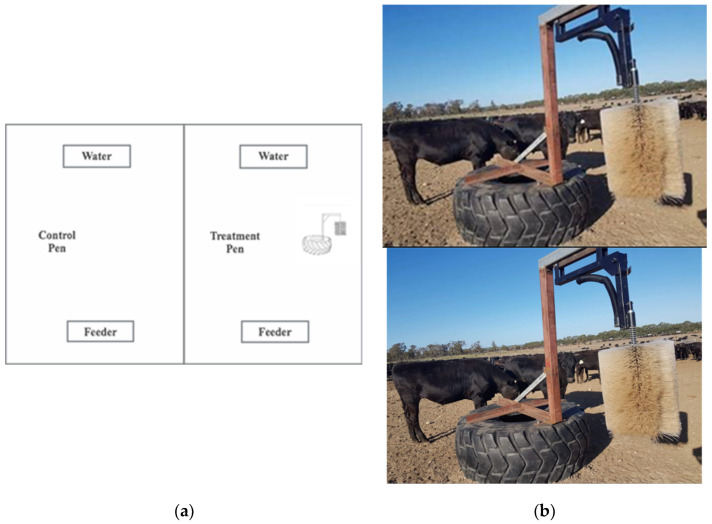
(**a**) Feedlot design and brush placement and (**b**) vertical grooming brush (Cowscratcher Complete, Redpath 2022).

The 169 Wagyu steers and heifers (14 months old) were bred and sourced from one property located in the south of Western Australia. Cattle were backgrounded in large paddocks on site for approximately three months prior to being placed within feedlot pens. This process occurred post-weaning and involved slowly introducing a grain diet, allowing social habituation, and administering appropriate treatments to ensure the cattle were healthy upon entering the feedlot pens. The cattle were allocated to one of two adjoined pens according to their starting bodyweights (light and heavy), which was commercial practice. Each pen was approximately 70 × 24 m (0.05 m^2^/head) with an electric perimeter fence and limestone base flooring. The pens were unshaded. Each pen had one self-feeder located at the higher end of the pen, at least 10 m from fence lines, and was refilled with ration every 2 to 4 days, depending on cattle appetite. Cattle were fed a grain ration, adjusted over the feeding program. This ration was designed by a nutritionist, with its composition adjusted over time to include appropriate levels of roughage and protein. These adjustments were made according to feed acclimatisation and the feedlot management schedules. An automatic concrete water trough was positioned at the lower end of each pen (Figure 1).

### 2.2. Treatments

A vertical grooming brush (Cowscratcher Complete, Redpath, Victoria, Australia) was fixed to a metal stand attached to a tractor tyre and positioned in the middle of one study pen (Figure 1). Eighty-nine cattle were assigned to the pen containing the enrichment brush (EB) and 80 cattle to the control pen (CON) without a brush. As the study was part of a commercial operation, allocation to pens was based on live weight by the property owner, with cattle being lighter in the EB group (433.7 ± 3.7 kg) than the CON group (507.6 ± 3.5 kg) on d1.

### 2.3. Behavioural Observations

A species-specific ethogram and a list of demeanour terms were used to assess behaviour at the pen level in both feedlot pens and behavioural interactions with the brush were recorded in the EB pen (Table 1). Demeanour was scored using the mobile phone application Kizeo Forms [36]. A list of descriptive terms was scored using a visual analogue scale (VAS) from 0 to 100 (0 indicates the term was not being expressed while 100 indicates the term was expressed to its fullest by the cattle). A detailed explanation of the VAS scoring system is provided by the Welfare Quality Network protocols [37].

Ethogram behaviour and demeanour were assessed on five days within the study period (d 2, 25, 50, 78, and 94) for an hour duration conducted at four time points within each day (0800 h, 1030 h, 1400 h, and 1600 h). At each of these time points, the posture and activity of CON and EB cattle was collected at a pen level by one observer using an ethogram and instantaneous scan-sampling, conducted every 5 min within one hour. The time spent with the brush was recorded for each individual interaction over an hour at each of the four timepoints. The duration of each interaction with the brush was summed for each hourly period.

The demeanour of the EB and CON cattle was recorded at a group level in the middle and end of each hour by a second observer. The cattle were viewed by the two observers from a car positioned approximately 15 m from the feedlot fence line. The nature of the behavioural assessment required observers to be aware of the treatment being administered. To help mitigate potential bias, the observers were experienced in ethogram and demeanour assessment and had received in-person training on scoring protocols prior to the study.

### 2.4. Production and Temperament Tests

The live weight and temperament of individual cattle were measured on d 1 and 107. While being weighed in the crush, cattle were given a crush score and subsequently, as they moved forward to exit the crush, an exit speed was measured manually using a stop-watch. The crush score was given between 1 (calm, no movement) and 5 (rearing, twisting body, or trying to escape) as described in Grandin [39]. The exit speed was the time (seconds) taken for an animal to exit the crush and pass a 2 m point in front of the crush.

### 2.5. Climate

The daily maximum and minimum temperature (°C), solar exposure (MJ ^–2^), and rainfall (mm) was recorded from the Myalup weather station (33.06° S, 115.41° E) on behaviour observation days and on two consecutive days prior to these days using archive data from the Bunbury weather station (33.36° S, 115.64° E).

### 2.6. Statistical Analysis

The average percentage of each ethogram behaviour was calculated for two time points within each hour (30 and 60 min) to align with the demeanour recordings in that hour. Ethogram scores were square root transformed for normality and homogeneity of error variance and time points were averaged as either AM (0830 h, 1030 h) or PM (1400 h, 1600 h). Repeated measures ANOVA was performed, including the treatment, time, and day, using Statistica, version 14.1.0 [40]. One time point (1030 h) was omitted from analysis on d 94 because the grooming brush was broken. Each behaviour was analysed separately and some behaviours were also combined for analysis, including self-groom total (self-groom + brush groom) and total groom (self-groom + allogroom + brush groom).

The demeanour scores were analysed using Principal Component (PC) analysis [40], with components that had eigenvalues greater than 1 and total variance greater than 12% being analysed further. PC data were non-parametric and were Box–Cox transformed prior to analysis. PC graphs were created using GraphPad Software, Version 10.3.0, September 2024 [41]. Repeated measures ANOVA was performed to test for variation between treatment, time points, and days. To describe the dimensions, terms that were above 75% of the highest coefficient value were chosen. Spearman’s rank correlation coefficient (rS) was used to examine correlations of PC 1, 2, and 3 scores with ethogram scores (n = 76), live weight change (n = 164), crush score (n = 164), and exit speed (n = 163).

The live weight difference (difference between individual d 1 and d 107 weight), crush score, and exit speed were log transformed. The live weight difference was compared between treatments using ANCOVA with d 1 live weight fitted as a covariate. The exit speed was compared between treatment and day using repeated measures ANOVA. As crush score data were non-parametric, they were analysed using a Wilcoxon test and Mann–Whitney U test including treatment and day. To provide context to behaviour assessments, climate data were presented graphically. Due to the limited nature of the climatic data, statistical comparison with the behaviour assessments was not carried out.

## 3. Results

### 3.1. Ethogram Behaviour

**Figure 2:**
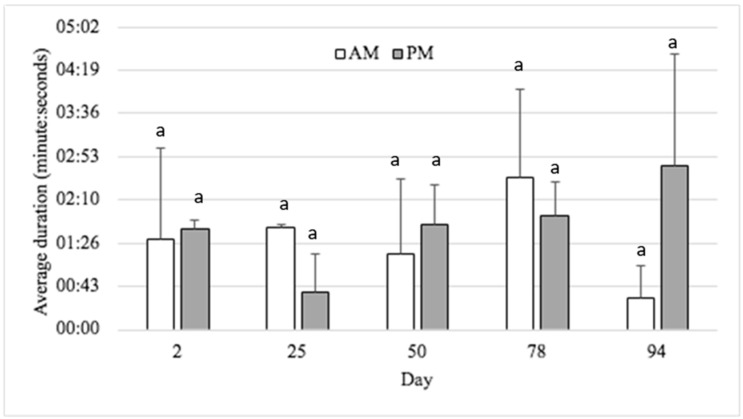
Average duration of time (minutes: seconds) cattle interacted with the enrichment brush in the EB pens during AM (0830 and 1030 h) or PM (1400 and 1600 h) timepoints on each study assessment day (±SE). Different letters indicate significant difference in time (AM or PM) and day.

A summary of the behavioural data comparing the treatments is presented in Table 2. The frequency of brush use and the duration of each brush interaction bout did not decrease over the 107 days that cattle were in the feedlot (Table 2). There were no meaningful differences in brush interaction between days or time of day; however, there was a spike in brush use following a three-hour period where the brush structure was removed for repair (d 94). The mean percentage of time cattle used the enrichment brush on each assessment day of the study ranged from 0.51 ± 0.28% to 2.01 ± 0.68% (Table 2). The average duration of interaction with the enrichment brush by cattle in the EB pen is shown in Figure 2; however, there was no difference over day or time of day. It was difficult to capture patterns in how cattle interacted with the enrichment brush over time as interactions were highly variable and frequently low in some behaviours. The assessment of the frequency of EB grooming by body region found that cattle predominantly used the EB to groom their face (34.12 ± 6.64%), followed by their rump (3.70 ± 1.97%) and then back (1.37 ± 0.61%) when averaged over days in the feedlot. Queuing to use the EB was observed in low frequency on d 2 and 50 (mean of those days 0.38 ± 0.13%).

Both the EB and CON cattle exhibited similar levels of self-grooming and allogrooming events. However, the additional grooming via the brush in the EB treatment group resulted in a higher total number of grooming events (self-grooming, allogrooming, and brush grooming) compared to the CON cattle (self-grooming and allogrooming only) (*p* < 0.05) (Table 2). Self-grooming and allogrooming events were higher in both EB and CON in the AM than PM time points (*p* < 0.05). Self-grooming was lower on d 2 than the other study days (*p* < 0.01) (Table 2). Allogrooming and self-grooming did not have an increasing trend over the study period, but d 94 had the highest frequency of both allogrooming (*p* < 0.01) and self-grooming (*p* < 0.05) compared to the other assessment days. Exploring behaviour was also higher on d 94 than all other study days (*p* < 0.05; Table 2).

**Figure 3:**
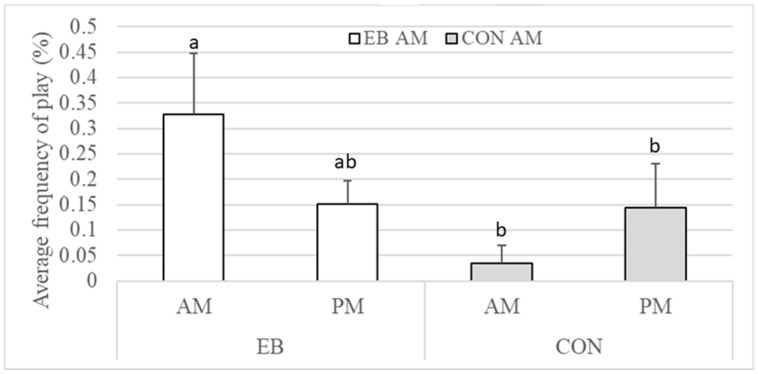
Average percentage (±SE) of play behaviours of cattle per treatment during AM (0830 and 1030 h) or PM (1400 and 1600 h) timepoints. Different letters indicate significant difference for treatment × time effects.

Cattle in the EB pen played more frequently in the morning (AM) compared to the afternoon (PM) and played more often in the morning (AM) than CON cattle at both AM and PM timepoints (*p* < 0.05, Figure 3). Aggression and abnormal behaviours were minimal and were not found to vary across days nor time of day. Both the EB and CON cattle were standing less earlier in the study (d 2) than at d 50, 78, and 94 (*p* < 0.05) and spent more time exploring later in the study (d 78 and 94) compared to earlier assessment days (*p* < 0.05; Table 2). On d 78, both treatment groups were more vigilant, ruminating less and resting less than other days in the study period (*p* < 0.01). They also spent less time lying down on d 78 compared to d 2, 25, and 50 and meaningfully less than on d 94 (Table 2). Both the EB and CON cattle had more drinking events on d 50 compared to d 2, 78, and 94 (*p* < 0.01) and spent more time eating in the PM time points (*p* < 0.05).

When the CON and EB treatments were averaged together for each behaviour over the study period it was found that the cattle spent most of their time stationary, mostly standing (71 ± 1.36% of time) rather than lying, with cattle displaying either resting (51 ± 1.39%) or alert behaviour (35 ± 2.55%). Cattle exhibited the three forms of grooming that included self-grooming (0.7 ± 0.05%), allogrooming (0.5 ± 0.04%), and brush grooming (1 ± 0.05%) in the EB pen only.

### 3.2. Demeanour

The assessment of demeanour found three PC dimensions that explained 67% of the total variance observed. For PC 1, the axis terms were *alert*, *curious,* and *active* at one end and *settled* at the other. For PC 2, the axis terms were *content* at one end, with no opposing term, while for PC 3, the term *anxious* was at one end and *sociable* at the other.

**Figure 4:**
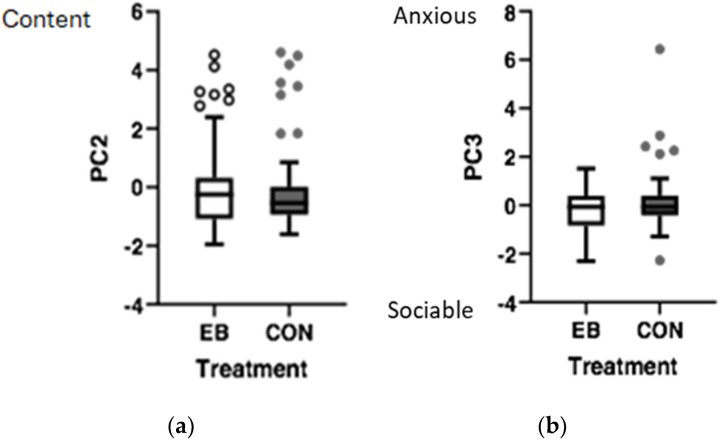
(**a**) PC 2 and (**b**) PC 3 scores of cattle housed in pens either with environmental enrichment brush (EB; open box/circles) or without (CON; closed box/circles).

Overall, the EB cattle were not different from the CON cattle on PC 1; however, both groups were scored as more *alert*, *curious,* and *active* on d 75 than on other measuring days. Overall, the mean PC 2 score for the EB cattle was slightly higher than that for the CON cattle (*p* < 0.05), indicating the EB cattle were scored as more *content* than the CON cattle (Figure 4a), particularly during the PM time points. Both the EB and CON cattle were also scored as more *content* on d 25 and 50 than on d 2 and 75 (*p* < 0.01) and on d 25 more than on d 94 (*p* < 0.05). Overall, the mean PC 3 score for the CON cattle was slightly higher than that for the EB cattle (*p* < 0.05), indicating the CON cattle were scored as more *anxious* than the EB cattle (Figure 4b), and the EB and CON cattle were scored as more *sociable* (PC 3) at the end of the study (d 94) (*p* < 0.01).

There were significant correlations of PC scores with ethogram and temperament measures; however, the correlations were low, warranting caution in assuming strong dependencies. PC 1 scores were positively correlated with standing (*β* = 0.328, *p* < 0.05), indicating that as more standing occurred cattle were described as more *alert*, *curious*, and *active*. Conversely, lying down was negatively correlated with PC 1, indicating that as this behaviour increased cattle were described as more *settled* (rS = −0.330, *p* < 0.01). There was a positive correlation of PC 2 scores with self-grooming (rS = 0.308, *p* < 0.01), total self-grooming, (rS = 0.271, *p* < 0.05) and positively engaged activities (rS = 0.254, *p* < 0.05), indicating that when cattle increased these behaviours they were described as more *content*. Exit speed was positively correlated with PC 3 (rS = 0.202, *p* < 0.05), showing that the cattle with a higher exit speed were described as more *anxious* while in the feedlot.

### 3.3. Live Weight and Temperament

Live weight increased in both the EB and CON treatment groups from d 1 to d 107 (*p* < 0.01) by an average of 96.7 ± 1.91 kg but did not differ between treatments (Table 3). Both the EB and CON treatment groups had an increased exit speed (*p* < 0.01) from d 1 to 107 (Table 3) but no treatment effect. Crush scores were higher in the CON than EB cattle on both d 1 and 107 (*p* < 0.01). Weight difference was positively correlated with exit speed (*β* = 0.232, *p* < 0.01). Exit speed was negatively correlated with crush score (*β* = −0.127, *p* < 0.05). There was no correlation of change in live weight, exit speed, and crush score to other ethogram measurements.

### 3.4. Climate

**Figure 5:**
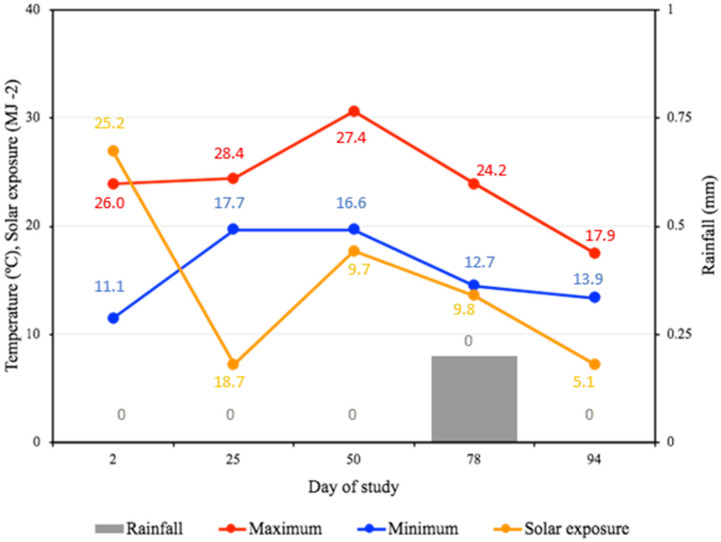
Daily maximum and minimum temperature, rainfall, and solar exposure (Mylup weather station) on study assessment days. Numbers indicate mean climate measures for the two consecutive days immediately prior to each of the study assessment days (Bunbury weather station).

The maximum daily temperature during the study period was an average of 25.1 ± 0.4 °C (range: 16.1 to 35.3 °C). Of the behavioural assessment days, d 50 had the highest daily maximum (30.6 °C); however, the 2 days prior to this were cooler (Figure 5). The night-time temperature dropped significantly during the study period to an average daily minimum of 13.0 ± 0.4 °C (range: 3.6 to 22.9 °C). The average daily solar exposure during the study period was 15.5 ± 6.2 MJ m^−2^ (range: 3.8 to 27.2 MJ m^−2^) and had a decreasing trend from summer (d 2) through the autumn months (d 25–94) (Figure 4). Daily rainfall was infrequent, with a maximum of 59 mm (d 60), but generally being 0 mm (86% of the study period). Day 78 was the only behavioural assessment day that recorded rainfall (Figure 5).

## 4. Discussion

Concern for cattle confined for extended periods can be addressed by providing a form of enrichment. Selecting an enrichment type that is species-appropriate and feasible for use under commercial conditions is a key consideration. In the current study, cattle demonstrated a sustained interest in the EB despite their 107-day confinement. The natural behaviour and demeanour assessment in this study provided insight into the positive welfare benefits of brush enrichment. This study found that provision of an enrichment brush did not influence live weight gain or temperament or result in more agonistic behaviour when compared to the CON treatment.

Cattle were observed to have a sustained interest in the provided brush. This corresponds with results from other studies examining the response of cattle to EB in a feedlot [12,14] and dairy management system [15,21,22]. In these studies, use was sustained over 6 to 64 days. The longer duration of the current study (107 days) corresponds to the duration of a feeding program typical of an Australian feedlot. The sustained use of the EB over this period suggests that it is a valuable form of enrichment in a feedlot context for providing long-term mental and physical stimulation. The question of whether this sustained use was the result of sustained individual frequency of use or different animals using the structure infrequently on different days was not assessed in this study. However, other studies have found that an EB was used by all feedlot cattle in the first 2 days of introduction [14] and by 97 to 99% of dairy cattle in the first week of introduction [13,21]. Furthermore, these studies and others have found that once familiar with this enrichment, cattle will use it daily, suggesting its novelty was not the main driver of interaction [5,42,43,44]. However, novelty may have a place in further promoting the use of an enrichment structure [45]. It is interesting to note that following the breakage and repair of the EB structure on d 94, there was a spike in frequency of its use. Although the repaired structure was returned in the same state prior to the breakage, the restriction of use during the repair period for around 3 h and reintroduction may have increased the subsequent interest in the structure. The periodic change or pen rotation of the EB could add value to its enrichment potential and use across a feedlot, where further studies could investigate the most efficient changes based on labour requirements and cattle usage response.

Cattle have a natural need to groom themselves and each other to maintain overall well-being and social bonds. They do this through self-grooming, allogrooming, and/or using objects for grooming hard-to-reach places [28,46,47]. The provision of the EB did not impact the frequency of other grooming methods, with no difference in frequency of self- grooming or allogrooming between treatments in the current study. Similar results were found in EB studies with grazing cattle [9,47], but other studies on dairy calves [28] and beef cattle [16] held at higher stocking rates in confined pens found an increase in self-grooming with the provision of a brush or other grooming devices. When self-grooming and allogrooming behaviours were combined, the cattle with access to the EB had a higher total grooming than the control cattle. It is suggested that self-grooming and allogrooming behaviour represent distinct behavioural needs independent from grooming on inanimate objects [19,47]. Kohari et al. [47] found that grooming on trees was a significant part to overall grooming behaviour in cattle. In feedlot pens, the absence of trees and natural grooming objects is common. Studies have shown that without grooming structures like brushes, cattle tend to rub against water troughs and pen walls [13]. In the current study, as well as in previous studies done on dairy cattle [3,21,22], the enrichment brush was mainly used for face grooming. However, Oliveira et al. [48] observed that dairy cattle spent the most time grooming their hindquarters, which was also the second most common use of the enrichment brush in the current study. Cattle likely used the brush for these areas because they could not reach them through self-grooming [13,14]. Consequently, using an enrichment brush can help reduce damage to facility infrastructure by providing a suitable grooming alternative for these hard-to-reach places.

Both self-grooming and allogrooming events were more frequent in the morning (AM) than in the afternoon (PM) across both treatment groups. This contrasts with Meneses et al. [49], who found that allogrooming in feedlot cattle was more common in the PM. Additionally, Meneses et al. [49] observed higher brush use in the PM, whereas in the current study, brush use was not significantly affected by the time of day. Interestingly, the timing of feeding events did not correlate with brush use. In the Meneses study [49], cattle were fed in the morning, with grooming events peaking in the afternoon. Conversely, in the current study, grooming events were more frequent in the morning, while eating events were higher in the afternoon across both treatment groups. Further research is needed to explore the relationship between grooming with an enrichment brush and eating, particularly in the feedlot context. Previous studies have shown that EB use decreases as its distance from the feed trough increases [50,51,52]. Mandel et al. [53] also found that lame cows preferred brushes located closer to the feed bunk, likely due to the reduced effort required to access them. In the current study, the EB was positioned halfway between the water and the feed trough. Future studies could investigate the optimal location of an EB in terms of welfare and productivity in different contexts, particularly if placing an EB near a feed trough encourages feeding events.

Good welfare is now recognized to extend to the presence of a positive affective state in an animal [54] and had not previously been examined in the context of enrichment brush use for livestock. The use of QBA and demeanour scoring allows interpretation of an animal’s affective state through assessment of body language and behavioural expression [54,55]. In the current study, cattle in the EB treatment were scored higher on PC 2, or were more *content*. Furthermore, both self-grooming and positively engaged ethogram behaviours were positively correlated with PC 2. Russell et al. [56] also found that dairy cattle were assessed as more *content*, *relaxed*, and *positively occupied* during periods of access to additional environmental resources (novel object and outdoor yard). Cows appearing more *content* in the EB treatment of the current study suggests they had a more positive affective state than those without the enrichment. In contrast, cattle without the EB were scored higher on PC 3, or were more *anxious*. The term *anxious* was not used in the study by Russell et al. [56], but *fearful* was a term negatively correlated on PC 1. During periods of access to additional environmental enrichment, cattle were scored as less fearful [56]. This term was also loaded with *bored*, with the authors highlighting the link between boredom, anxiety, and fear found in human studies [57] and anxiety-related behaviours in rats with reduced environmental enrichment [58,59]. It was suggested that increasing animals’ time in positively engaging activities such as enrichment would likely decrease time spent in boredom—like situations where cows are more aware of their surroundings and potential threats [56]. The results from the current study support the need of cattle to have a more complex environment through enrichment in feedlot production systems, which are often barren and lack stimulation.

Studies have found that provision of an EB reduces handling stress by observing a decrease in heart rate [13,44]. In the current study, cattle described as being more *anxious* also had a higher exit speed (or less calm temperament). Curley et al. [60] found that the way cattle experience stress is influenced by the animals’ temperament and that their physiological stress responses associated with temperament persist throughout the lifetime of cattle. This highlights the importance of enrichment, in this case through a brush, for cattle with less calm temperaments that are prone to these increased stress responses. Lower stress responses are crucial in improving overall health, enhancing meat quality, and ensuring safer interactions between producers and their livestock [61].

Cattle enriched with the EB were scored lower on PC 3, or were more *sociable*. Play-based ethogram behaviour, although not correlated with PC 3 in this study, would likely be viewed as an element of sociable body language. Play behaviour was found to be more frequent in the cattle exposed to the EB compared to the CON cattle. In most cases, play behaviour is associated with improved welfare and is easily blocked by detrimental environmental conditions [62]. A study on EB use in beef cattle also found that exposure to EB increased play behaviour [27]. This finding and those in the present study suggest that the provision of an enrichment brush not only enhances sociability in cattle but also promotes play behaviour, which is indicative of improved welfare.

PC 1 was characterised by *curious*, *alert*, and *active* at one end and *settled* on the opposing axis. This dimension was positively correlated with standing behaviour in the cattle. The PC terms suggest that PC 1 is associated with activity level in the cattle, similar to studies on cattle and sheep penned in small groups during transport [63,64] and paddock-penned cattle [65]. The lack of treatment significance on this dimension suggests activity did not explain behavioural expressions related to access to the brush enrichment. This could be related to the context of the study, where cattle were acclimated to the feedlot prior to study commencement, possibly reducing the curiosity and heightened activity or liveliness associated with a novel environment [45]. Acclimation may also explain the low frequency of agonistic behaviours seen in the current study. Tennessen et al. [66] found agonistic behaviours often reduce after 10 days as cattle become more familiar with their cohort and surroundings. It is positive that agonistic encounters of EB cattle were not higher early in the study (d 2) when the EB was novel. Reyes et al. [15] found similar results to the current study where displacement of cattle from an EB and competition for it were low. Queuing for use of the EB was also observed in the current study and was also found in a study by Dickson et al. [9] on pasture beef cattle response to an EB. This suggests that producers can provide such an enrichment and expect low levels of aggression and injuries associated with resource guarding. It also suggests that the provision of only one brush for a group of cattle of up to 90 cattle is practical, making the provision of such enrichment more feasible and cost-effective.

The cattle in the current study exhibited a lack of abnormal and therefore stereotypic behaviour. Stereotypies can be defined as maladaptive or atypical behaviours, often repetitive and periodic in nature, which are considered to arise from prolonged frustration [67]. Frustration occurs when animals are unable to express highly motivated and natural behaviours [3]. Although the term ‘*frustration*’ was included in the term list, it did not load on any PC axis in this study. The absence of cattle observed to be frustrated and/or observed to be performing stereotypic behaviour in the study supports the effectiveness of the feedlot management system. Research indicates that intensive management systems can limit an animal’s ability to express natural behaviour [68]. Additionally, the Wagu breed, known for its docile nature and low aggression [69], may have contributed to the low incidence of agonistic behaviour observed in this study. Previous feedlot cattle studies on different beef breeds have recorded much higher agonistic and stereotypic behaviours than those observed current study [14,70,71,72].

Enrichment is important in improving cattle welfare and promoting natural behaviours, with short-term productivity gains not being the primary focus of enrichment implementation. Although enrichment may not improve productivity, it is important that it does not diminish it, as maintaining productivity ensures the sustainability and economic viability of the production system. No difference in weight gain between treatment groups was found in the current study, with the small sample size and the confounding effects of initial weight difference between treatments making any data interpretation difficult. However, other studies have also shown no impact of EB access on weight gain in beef cattle [14,16]. Despite EB not improving live weight gain, the current study showed that the EB did not negatively influence it.

Although the climate in this study was well above the thermoneutral zone on some days [73], this was not sustained for consecutive days. The temperature also decreased at night, so the heat load was not accumulated [74]. The hottest day of the study (d 50) showed a higher frequency of drinking compared to other days. There was also a significant jump in vigilance and decrease in rumination and resting behaviour on d 78. This was the only assessment day with rainfall recorded. Anecdotal reports were that the cattle appeared to have high energy and the day was reflective of a ‘cool change’. Studying cattle production over a long period with varied weather conditions will help develop an understanding of how these conditions influence cattle productivity and welfare over time [75]. The chronic exposure of cattle to heat load is often present within Australian feedlot production systems during summer [3]. Exposure of cattle to a high Thermal Heat Index has been shown to decrease EB use in a study by Mandel et al. [50]. The current study only encompassed the last 2 weeks of summer and therefore the influence of enrichment during periods of heat load within a feedlot context needs further study.

The current study aimed to limit confounding factors to the hypotheses while, importantly, minimising the influence of the study on the commercial management of the feedlot to allow comparison within this context. These presented challenges related to available infrastructure and limited pen replicates. The large group size did not allow for the individual identification of all the animals in this study. Studies on individual variation may be limited to smaller, more controlled studies or RFID sensor technology. Studying the cattle as a group, rather than a selection of focal animals, offered advantages given the limited pen replicates and better managed the potential confounding factor of initial live weight differences between treatment groups. By examining animals as a group, we can better detect all signs of welfare, avoiding missed interactions and distress in non-focal animals that might be outliers in terms of live weight and other characteristics and accounting for varied stress responses.

Difference in initial temperament (measured by crush score) between treatment groups could have also presented a confounding influence on cattle behaviour. Interestingly, flight speed did not differ between groups on day 1. Gibbons et al. [76] found discrepancies between flight speed and crush score in docile dairy breeds, which typically had crush scores of 1 to 1.5. They suggested that a crush score of 1 might include both tame and very fearful animals, as both tended to stand still. Because the management of lot–fed cattle is carefully controlled to optimise production, further research should ensure that any enrichment strategies do not compromise this. For example, diets are carefully formulated and controlled to maximise production and providing nutritional enrichment is often not practical. Further studies could investigate the effectiveness and integration of medication delivery systems into enrichment brushes to improve cattle health and welfare, possibly leading to improved production. Existing products, such as oil-dispensing grooming brushes for fly and lice control [77], demonstrate the potential for such innovations.

## 5. Conclusions

The current study aimed to evaluate the use of an enrichment brush by beef cattle in a commercial feedlot whilst minimising any influence on the management of the feedlot. Notably, brush use was maintained throughout the feedlot period, suggesting it provides long-term benefit to the animals. Furthermore, agonistic behaviours were not different between treatments, suggesting that the enrichment did not promote competition between animals. The lack of agonistic encounters is positive in terms of it representing a reduced risk of injury and therefore carcass bruising, in addition to reducing damage to facility infrastructure. The natural behaviours and affective state of the EB cattle provided some insight into the positive effect of brush enrichment on the animals’ welfare. Brush enrichment helps address societal concerns of the barren nature of feedlot production systems by providing cattle with a more stimulating and engaging environment. Higher welfare does not necessarily equate to higher production, but production (as measured by live weight) was not compromised with the implementation of the brush enrichment in the current study. The results of this study show that the implementation of an enrichment brush can improve the natural behaviour and affective state of cattle within a feedlot context without compromising productivity. Therefore, brushes are a practical and feasible form of enrichment that feedlots could adopt. This study highlights the importance of enrichment for cattle within a feedlot system for a more ethically sustainable industry.

## Figures and Tables

**Table 1:** Behaviours and demeanour terms used to assess cattle during the feedlot period.

Ethogram behaviours (see Petherick et al. [38] for full definitions of terms)	Standing, lying (sternal or lateral), walking, resting, ruminating, vigilant, eating, drinking, aggression (mount, head butt, kick or charge), abnormal behaviour (tongue rolling, bar/fence chewing), play, push, self-grooming, brush-grooming, allogrooming, explore
Behaviours associated with the brush or tyre in treatment pen	Time spent with brush (seconds), frequency of use, sniffing (brush or tyre), total grooming (face (brush or tyre), rump or back) on structure, chewing (brush or tyre), displacement, queuing for access to structure, aggression, playing with structure
Demeanour scoring terms (see Taylor et al. [32] for full definitions of terms)	Active, agitated, alert, content, curious, dull, frustrated, settled

**Table 2:** Mean percentage (±SE) that ethogram behaviours were exhibited by cattle while held in pens either with environmental enrichment brush (EB) or without (CON) on each study assessment day.

Treatment	Behaviour	Day				
		2	25	50	78	94
Standing ^	EB	49.41 ± 12.65	66.78 ± 9.55	69.73 ± 6.10	93.65 ± 2.23	71.72 ± 13.03
	CON	51.82 ± 11.26	61.88 ± 6.71	76.98 ± 5.60	89.43 ± 4.04	76.88 ± 14.92
Resting ^	EB	69.41 ± 11.37	71.77 ± 4.28	48.20 ± 8.73	13.07 ± 5.04	84.79 ± 5.36
	CON	71.67 ± 4.77	64.14 ± 2.94	49.58 ± 8.98	2.16 ± 0.91	38.51 ± 13.69
Vigilant ^	EB	20.53 ± 10.79	15.38 ± 3.86	37.27 ± 8.23	73.56 ± 3.88	4.97 ± 1.63
	CON	15.75 ± 4.38	25.94 ± 2.33	36.07 ± 7.19	83.93 ± 2.94	40.80 ± 9.56
Eating #	EB	0.98 ± 0.50	1.66 ± 0.60	2.93 ± 1.11	4.48 ± 1.38	6.60 ± 2.17
	CON	2.00 ± 1.02	1.30 ± 0.69	4.35 ± 2.08	2.84 ± 1.03	5.31 ± 2.08
Drinking ^	EB	0.84 ± 0.28	1.87 ± 0.27	1.92 ± 0.18	0.63 ± 0.17	0.76 ± 0.46
	CON	1.12 ± 0.29	2.06 ± 0.27	2.63 ± 0.30	0.70 ± 0.13	1.32 ± 0.44
Ruminating ^	EB	4.75 ± 0.84	5.52 ± 0.79	5.10 ± 0.53	1.19 ± 0.28	5.28 ± 0.60
	CON	7.91 ± 1.37	4.51 ± 0.90	4.92 ± 0.52	1.77 ± 0.25	4.31 ± 1.42
Playing *#	EB	0.09 ± 0.05	0.21 ± 0.16	0.23 ± 0.16	0.26 ± 0.09	0.42 ± 0.23
	CON	0.02 ± 0.02	0.03 ± 0.03	0	0	0.52 ± 0.26
Explore ^	EB	0.16 ± 0.07	0.75 ± 0.23	0.80 ± 0.22	2.93 ± 0.83	1.77 ± 0.61
	CON	0.03 ± 0.03	0.21 ± 0.08	0.26 ± 0.13	2.73 ± 0.10	3.78 ± 2.21
Allogroom ^	EB	0.12 ± 0.06	0.63 ± 0.21	0.44 ± 0.18	0.10 ± 0.07	1.11 ± 0.30
	CON	0.26 ± 0.15	0.39 ± 0.16	0.18 ± 0.09	0.13 ± 0.08	1.42 ± 0.33
Self-groom ^	EB	0.05 ± 0.03	0.82 ± 0.24	0.66 ± 0.17	0.68 ± 0.18	1.08 ± 0.29
	CON	0.10 ± 0.05	0.78 ± 0.23	0.65 ± 0.13	0.63 ± 0.28	1.88 ± 0.44
Brush groom		0.84 ± 0.32	0.51 ± 0.28	1.05 ± 0.37	0.73 ± 0.21	2.01 ± 0.68

Differences were considered statistically significant at *p* < 0.05 and effects within 0.05 < *p* < 0.10 were considered meaningful tendencies. * indicates difference between EB and CON treatment at *p* < 0.05. ^ indicates day effect at *p* < 0.05. # Indicates meaningful tendencies of effect of day (0.05 < *p* < 0.10).

**Table 3:** Live weight, crush score, and exit speed of cattle housed in pens either with environmental enrichment brush (EB) or without (CON) at the beginning (d 1) and end of study (d 107). All values are presented as mean ± standard error. Different letters indicate significant differences *p* < 0.05.

	CON		EB	
	d 1	d 107	d 1	d 107
Live weight (kg)	507.61 ± 3.50 ^a^	607.12 ± 4.70 ^b^	433.72 ± 3.74 ^a^	527.79 ± 5.15 ^b^
Crush score (1–5)	1.70 ± 0.06 ^a^	1.82 ± 0.06 ^a^	1.49 ± 0.06 ^b^	1.51 ± 0.03 ^b^
Exit speed (seconds)	3.42 ± 0.12 ^a^	3.99 ± 0.15 ^b^	3.20 ± 0.10 ^a^	3.62 ± 0.12 ^b^

## Data Availability

The datasets presented in this article are not readily available because of commercial sensitivity. Requests to access the datasets should be directed to the corresponding author.
